# Reactive Oxygen Species Yield near Gold Nanoparticles Under Ultrahigh-Dose-Rate Electron Beams: A Monte Carlo Study

**DOI:** 10.3390/nano15171303

**Published:** 2025-08-23

**Authors:** Chloe Doen Kim, James C. L. Chow

**Affiliations:** 1 Radiation Medicine Program, Princess Margaret Cancer Centre, University Health Network, Toronto, ON M5G 1X6, Canada; chloe.kim@uhn.ca; 2 Department of Radiation Oncology, University of Toronto, Toronto, ON M5T 1P5, Canada; 3 Department of Materials Science and Engineering, University of Toronto, Toronto, ON M5S 3E4, Canada

**Keywords:** gold nanoparticles, FLASH radiotherapy, reactive oxygen species, Monte Carlo simulation, ultrahigh dose rate, electron beam, nanoparticle-enhanced radiotherapy, dose enhancement, yield enhancement factor, DNA damage, DNA dosimetry, nanodosimetry

## Abstract

Ultrahigh dose rate (UHDR) radiotherapy, also known as FLASH radiotherapy (FLASH-RT), has shown potential for increasing tumor control while sparing normal tissue. In parallel, gold nanoparticles (GNPs) have been extensively explored as radiosensitizers due to their high atomic number and ability to enhance the generation of reactive oxygen species (ROS) through water radiolysis. In this study, we investigate the synergistic effects of UHDR electron beams and GNP-mediated radiosensitization using Monte Carlo (MC) simulations based on the Geant4-DNA code. A spherical water phantom with embedded GNPs of varying sizes (5–100 nm) was irradiated using pulsed electron beams (100 keV and 1 MeV) at dose rates of 60, 100, and 150 Gy/s. The chemical yield of ROS near the GNPs was quantified and compared to an equivalent water nanoparticle model, and the yield enhancement factor (YEF) was used to evaluate radiosensitization. Results demonstrated that YEF increased with smaller GNP sizes and at lower UHDR, particularly for 1 MeV electrons. A maximum YEF of 1.25 was observed at 30 nm from the GNP surface for 5 nm particles at 60 Gy/s. The elevated ROS concentration near GNPs under FLASH conditions is expected to intensify DNA damage, especially double-strand breaks, due to increased hydroxyl radical interactions within nanometric distances of critical biomolecular targets. These findings highlight the significance of nanoparticle size and beam parameters in optimizing ROS production for FLASH-RT. The results provide a computational basis for future experimental investigations into the combined use of GNPs and UHDR beams in nanoparticle-enhanced radiotherapy.

## 1. Introduction

Radiotherapy remains a cornerstone in the treatment of various malignancies, and recent advances have aimed to enhance its efficacy while minimizing damage to healthy tissues. One such advancement is FLASH radiotherapy (FLASH-RT), which involves the delivery of radiation at ultrahigh dose rates (UHDRs), commonly defined as dose rates exceeding 40 Gy/s. This emerging modality has demonstrated the potential to achieve comparable or superior tumor control compared to conventional dose rates while significantly reducing normal tissue toxicity [1,2,3]. The so-called FLASH effect, a phenomenon wherein normal tissues experience enhanced sparing without compromising tumor control, has been supported by multiple preclinical studies and is currently under active investigation for clinical translation [4].

Several hypotheses have been proposed to explain the mechanisms underlying the FLASH effect. One prevailing theory involves transient oxygen depletion in normal tissues during UHDR exposure, which induces a temporary hypoxic state that renders the tissues more resistant to radiation damage [4,5,6]. Early studies on bacteria [7] and mammalian cells [8] have laid the foundation for the oxygen depletion hypothesis [9]. Another theory posits a differential biochemical response between normal and cancerous tissues. In this framework, cancer cells, which often have impaired antioxidant defenses, are more vulnerable to oxidative damage induced by ionizing radiation. Conversely, normal cells may exhibit higher resistance to reactive oxygen species (ROS) due to more robust antioxidant systems. Moreover, the limited diffusion of ROS produced during water radiolysis at UHDR may further contribute to the selective targeting of cancerous tissues [5,10]. It is also noted that FLASH-RT can reduce inflammation in normal tissues while enhancing anti-tumor immunity, preserving a greater number of circulating immune cells to survive compared to conventional radiotherapy [4,9].

Preclinical studies using various radiation modalities and delivery techniques have demonstrated that FLASH-RT can significantly spare normal tissues while maintaining therapeutic efficacy against tumors [11,12,13,14]. These findings underscore the promise of FLASH-RT as a paradigm-shifting approach in cancer therapy and have catalyzed interest in complementary strategies that further enhance its selectivity and potency [15].

In parallel, the application of engineered nanomaterials (ENMs), notably gold nanoparticles (GNPs), has gained substantial attention in the field of radiation oncology. According to the International Organization for Standardization (ISO), ENMs are materials with at least one dimension in the range of 1 to 100 nm [16,17]. Among them, GNPs have emerged as a particularly attractive class of radiosensitizers due to their biocompatibility, ease of surface modification, and high atomic number (Z = 79), which enhances their ability to absorb radiation [18,19]. Importantly, GNPs are generally considered safer for in vivo applications compared to other high-Z materials, making them suitable for clinical development [18].

Mechanistically, GNPs enhance the biological effect of radiation through both physical and chemical pathways. Physically, they increase local dose deposition via secondary electron emission. Chemically, the interaction of radiation with GNPs in an aqueous environment leads to enhanced ROS production, which amplifies oxidative stress and contributes to DNA damage in cancer cells [19,20,21,22,23,24]. Specifically, the photoelectric effect plays a dominant role in energy deposition when high-*Z* materials like gold interact with low-energy photons or electrons. In this process, incident radiation transfers energy to inner-shell electrons of gold atoms, ejecting photoelectrons and initiating a cascade of Auger electrons. These low-energy electrons travel short distances in water, leading to dense ionization tracks that cause water radiolysis and the subsequent formation of ROS (e.g., OH•, H_2_O_2_, H•). The localized production of these species near DNA significantly enhances the probability of inducing double-strand breaks (DSBs) and other lethal lesions [25]. Among the various forms of radiation-induced DNA lesions, DSBs are considered the most lethal and are a critical determinant of radiotherapeutic efficacy [20]. The potential benefit of GNP through enhanced ROS-induced DNA damage brings new prospects for personalized and targeted cancer treatments as nanotechnology in medicine progresses [26].

Despite significant research into the individual effects of UHDR radiation and GNP-mediated radiosensitization, there are limited studies that explore their combined interaction. This gap highlights the need for a deeper understanding of how GNPs influence ROS dynamics and DNA damage under FLASH-RT conditions. Monte Carlo (MC) simulation techniques offer a powerful tool for investigating these interactions at the nanoscale. In particular, Geant4-DNA, an extension of the Geant4 toolkit developed at the European Organization for Nuclear Research (CERN), provides a versatile platform for simulating radiation interactions with matter at both the physical and chemical stages, including the generation and diffusion of ROS in water [27,28].

Previous MC studies have examined dose enhancement ratios and energy deposition around GNPs using various radiation types, including photons [29,30,31,32], electrons [33], and protons [34,35]. In addition, the dependence of ROS enhancement on GNP size has been analyzed with different radiation modalities [35,36]. However, these studies primarily focus on conventional dose rates and do not account for the unique characteristics of UHDR delivery. To the best of our knowledge, few simulation studies have addressed the interplay between GNPs and FLASH-RT, particularly with respect to ROS production during water radiolysis at UHDR.

To address this gap, the present study employs Geant4-DNA-based MC simulations to investigate the influence of GNPs on ROS yield under electron-beam irradiation at UHDR. By modeling the spatial distribution of ROS and comparing it to an equivalent water nanoparticle (WNP) control, we aim to quantify the yield enhancement factor (YEF) as a function of GNP size, dose rate, and distance from the nanoparticle. These insights are expected to provide computational support for experimental studies and help optimize nanoparticle-enhanced FLASH-RT strategies. Previous experimental studies have validated the enhanced production of ROS and increased DNA damage in the presence of GNPs under irradiation, supporting the predictions made by MC-based models. For example, Misawa and Takahashi [21] demonstrated increased ROS generation with GNPs under X-ray and UV exposure, while Butterworth et al. [22] and Hainfeld et al. [23] reported enhanced cytotoxicity and tumor control due to nanoparticle radiosensitization. These findings align with simulation outcomes and underscore the relevance of using MC tools like Geant4-DNA to model nanoscale radiation interactions and optimize radiotherapeutic conditions.

## 2. Materials and Methods

### 2.1. MC Simulation Using Geant4-DNA

MC simulations were employed in this study to model the interactions between incident electrons and biological media, including the presence of GNPs. The simulations were conducted using Geant4-DNA, an extension of the general-purpose Geant4 toolkit developed by CERN. Geant4-DNA is specifically designed to simulate the physical, physicochemical, and chemical interactions of ionizing radiation with liquid water at nanometric spatial resolutions, making it particularly well-suited for nanoscale radiobiology studies [37,38].

The simulations were performed using Geant4 version 11.2, which includes an updated chemistry module capable of modeling time-resolved radiolysis processes [35]. The Livermore physics model was adopted to describe particle interactions within the GNP due to its accuracy in representing electromagnetic interactions for high-Z materials at low energies. For the surrounding water medium, the Geant4-DNA physics models were applied to simulate the full cascade of interactions, including energy deposition, track structure, and chemical stage evolution of reactive species.

The simulations aimed to model a GNP embedded within a water environment under UHDR electron-beam irradiation. The system was carefully configured to enable the analysis of both physical dose deposition and chemical yield of ROS around the nanoparticle, providing a multiscale understanding of radiation-induced effects. The chemical stage of the simulation, including ROS formation, was implemented using the Geant4-DNA chemistry module, which models the time evolution of reactive species following water radiolysis. After energy deposition by primary and secondary electrons, the simulation proceeds through physicochemical processes, such as ionization, excitation, and dissociation, leading to the formation of early species (e.g., H_3_O^+^, e^−^_aq_, OH•, H•, and H_2_O_2_). These species are then tracked for up to 10 nanoseconds to account for diffusion and chemical reactions in the aqueous medium. ROS yield was quantified at this time point, consistent with literature indicating peak biological relevance of radical interactions within this timescale [3,39].

### 2.2. Simulation Geometry and Configuration

To approximate a simplified biological environment, the simulation geometry was designed as a spherical water phantom with a radius of 5 μm, large enough to ensure that secondary electrons and ROS products did not escape the simulation volume yet small enough to maintain computational efficiency [3]. At the center of the sphere, a spherical GNP was embedded, with diameters varied among 5, 10, 50, and 100 nm to study the size-dependent effects.

Incident electron beams were simulated to irradiate the GNP at two different initial energies: 100 keV and 1 MeV. These energy values were selected to approximate the local energy of electrons reaching tumor cells, after accounting for the attenuation and energy loss of clinical electron beams (typically 6–20 MeV) as they traverse tissue. At the cellular or subcellular level, particularly in the presence of GNPs, electrons in the range of 100 keV to 1 MeV are representative of the actual energy spectrum responsible for ionization and ROS production. Therefore, using these energies enables simulation of clinically relevant FLASH RT effects at the nanoscale. Each electron was emitted from the inner surface of the water sphere, directed toward the GNP center. The initial source position for each electron was randomly sampled from the water sphere’s surface, mimicking an isotropic irradiation field.

**Figure 1:**
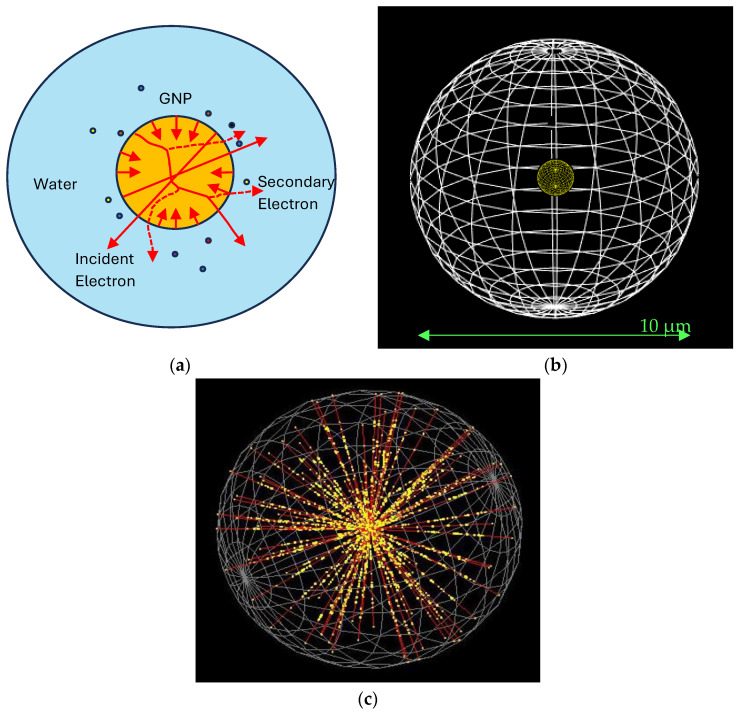
MC geometry modeled in Geant4-DNA. (**a**) A GNP (orange), with diameters of 5, 10, 50, or 100 nm, is positioned at the center of a water sphere (light blue) with a radius of 5 μm. ROS, incident electrons, and secondary electrons are denoted by multicolored dots, solid red lines, and dashed red lines, respectively. (**b**) The experimental setup for the MC simulation consists of the spherical water phantom (diameter of 10 µm) embedded with the GNP in varying size. (**c**) Electrons with an energy of 1 MeV are directed toward the center of the sphere from the surface of the GNP. Secondary electrons (yellow dots), generated along the electron tracks (solid red lines), deposit energy within the water sphere. The primary electron beams used in this simulation model the clinical delivery of electron-beam therapy under UHDR conditions. When these incident electrons interact with the GNPs and surrounding water medium, they undergo inelastic scattering, leading to the emission of low-energy secondary electrons. These secondary electrons are primarily responsible for inducing localized ionization and excitation in water molecules, resulting in the production of ROS through radiolysis. This cascade of electron-induced processes forms the mechanistic basis for radiosensitization in the presence of GNPs.

The simulation geometry is illustrated in Figure 1, where electrons (solid red lines) with initial energies of 100 keV or 1 MeV are irradiated toward the center of the water sphere from the surface of the GNP, as shown in Figure 1a. The experimental setup for the MC simulation is shown in Figure 1b, with the spherical water phantom (diameter of 10 µm) embedded with the GNP in varying size (a diameter of 5, 10, 50, and 100 nm). Secondary electrons (red dashed lines) generated from interactions within the GNP, along with ROS represented by multicolored dots, are also visualized. As these secondary electrons propagate along their tracks (solid red lines), they deposit energy into the surrounding water medium, initiating radiolysis, as depicted in Figure 1c.

### 2.3. Simulation of UHDR Electron-Beam Irradiation

The simulation of UHDR conditions was based on the characteristics of the Oriadtron eRT6 linear accelerator (PMB, Rousset, France), which delivers pulsed electron beams at clinically relevant UHDR parameters [40,41]. Each radiation pulse has a duration of 2 μs with a repetition rate of 100 Hz and a maximum dose delivery of up to 10 Gy per pulse.

The MC simulation used a single pulse out of 100 pulses in a second (100 Hz repetition rate). To realistically model these UHDR conditions in the MC framework, a methodology adapted from Chappuis et al. [3] was employed. During the simulation, electrons were propagated through the water sphere, and the total number of incident electrons was dynamically adjusted such that the deposited dose matched one of three target thresholds: 0.6, 1.0, and 1.5 Gy per pulse. These correspond to dose rates of 60, 100, and 150 Gy/s, respectively, which fall within the FLASH regime. The beam parameters used are summarized in Table 1.

This configuration allowed the investigation of dose rate-dependent effects on energy deposition and ROS production around the GNP under conditions that replicate preclinical FLASH-RT delivery.

### 2.4. ROS Quantification and YEF

To quantify the radiosensitizing impact of GNPs under UHDR irradiation, the formation of ROS was analyzed during the chemical stage of water radiolysis. In this phase, short-lived radicals, such as hydroxyl radicals (OH•), play a critical role in biological damage mechanisms, particularly in the formation of DNA DSBs [42].

According to prior studies, the diffusion and interaction of OH• radicals with biomolecules occur predominantly within the first 10 nanoseconds following radiation exposure. Beyond this window, ROS concentrations tend to saturate, and further reactions become secondary. Therefore, ROS concentrations were evaluated at 10 ns to capture the peak biological relevance of ROS-induced damage.

To isolate the effects attributable to GNP radiosensitization, a control simulation was also conducted using a WNP of identical size and position, replacing the GNP within the same simulation geometry. The YEF was then calculated as the ratio of total ROS yield, comprising H_3_O^+^, OH•, H_2_O_2_, OH^−^, and H• radicals, produced with GNPs to that with WNPs under identical conditions as shown in Equation (1).

(1) $$YEF=\frac{{ROS}_{GNP}}{{ROS}_{WNP}}$$

This metric provides a direct measure of the chemical amplification attributable to GNP radiosensitization under FLASH conditions and enables comparative analysis across different nanoparticle sizes, electron energies, and dose rates.

## 3. Results

### 3.1. Influence of UHDR on YEF

**Figure 2:**
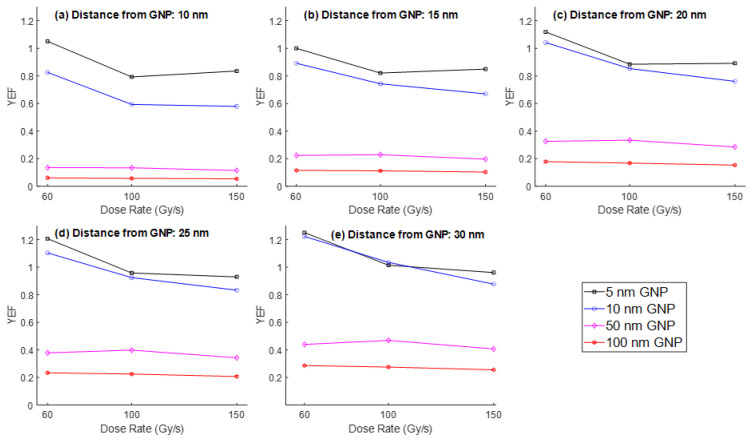
YEF vs. UHDR (60, 100, and 150 Gy/s) at distances of (**a**) 10 nm, (**b**) 15 nm, (**c**) 20 nm, (**d**) 25 nm, and (**e**) 30 nm from the surface of GNPs of different diameters (5, 10, 50, and 100 nm) for the electron energy of 1 MeV.

The effects of different UHDR, namely, 60, 100, and 150 Gy/s, on the YEF were evaluated at varying distances (10–30 nm) from the surface of GNPs of different diameters (5, 10, 50, and 100 nm). Simulations were conducted using two electron energies of 1 MeV and 100 keV. For 1 MeV electrons, the YEF was observed to be highest for small GNP sizes (5 nm and 10 nm) and low UHDR conditions (60 Gy/s). As shown in Figure 2, the YEF increased with increasing distance from the nanoparticle surface, reaching a maximum value of 1.25 at 30 nm from a 5 nm GNP at 60 Gy/s. While there was no change in YEF with an increase in UHDR from 100 Gy/s to 150 Gy/s in a 5 nm GNP, a decrease in YEF was found for the same UHDR change in a 10 nm GNP. This suggests enhanced ROS production farther from the GNP under specific conditions of lower dose rates and smaller particle sizes.

**Figure 3:**
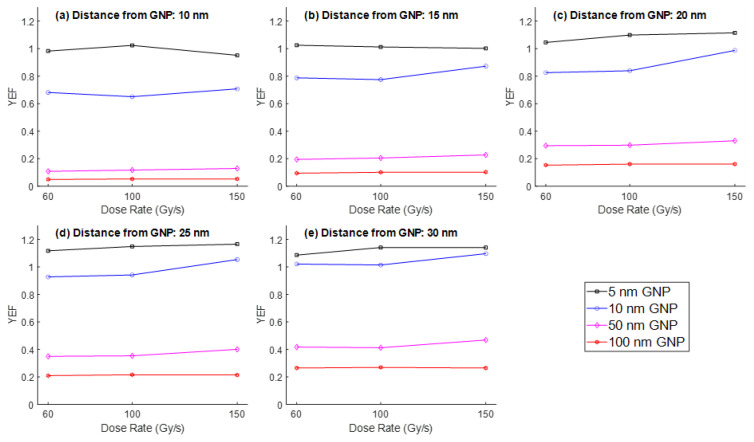
YEF vs. UHDR (60, 100, and 150 Gy/s) at distances of (**a**) 10 nm, (**b**) 15 nm, (**c**) 20 nm, (**d**) 25 nm, and (**e**) 30 nm from the surface of GNPs of different diameters (5, 10, 50, and 100 nm) for the electron energy of 100 keV.

In contrast, for larger GNPs (50 nm and 100 nm), the YEF remained relatively constant across all dose rates, indicating a reduced sensitivity to UHDR in terms of ROS amplification. When examining the results for 100 keV electrons (Figure 3), the dose rate had minimal influence on YEF across most GNP sizes. A slight increase in YEF was noted only in the 10 nm GNP case, where the highest YEF was recorded at 150 Gy/s. However, the overall enhancement was less pronounced compared to the 1 MeV case, and maximum YEF values remained below those obtained with high-energy electrons. Nevertheless, there was also a trend similar to 1 MeV where the YEF increased with increasing distance from the nanoparticle surface.

### 3.2. Effect of GNP Size on YEF

**Figure 4:**
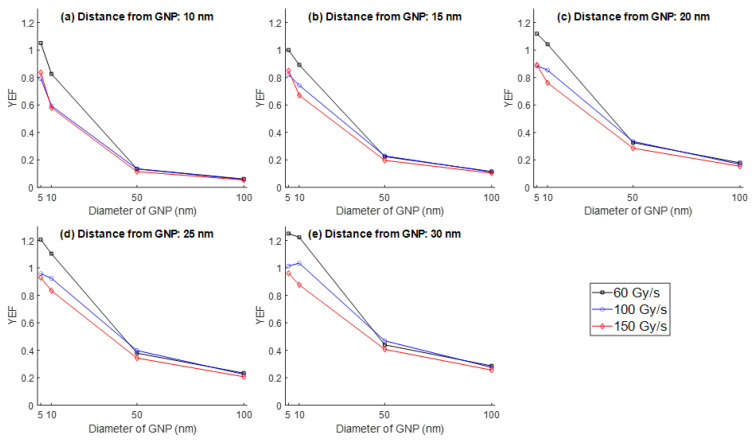
YEF vs. GNP size (5, 10, 50, and 100 nm) at distances of (**a**) 10 nm, (**b**) 15 nm, (**c**) 20 nm, (**d**) 25 nm, and (**e**) 30 nm from the surface of GNP at different UHDR (60, 100, and 150 Gy/s) for the electron energy of 1 MeV.

**Figure 5:**
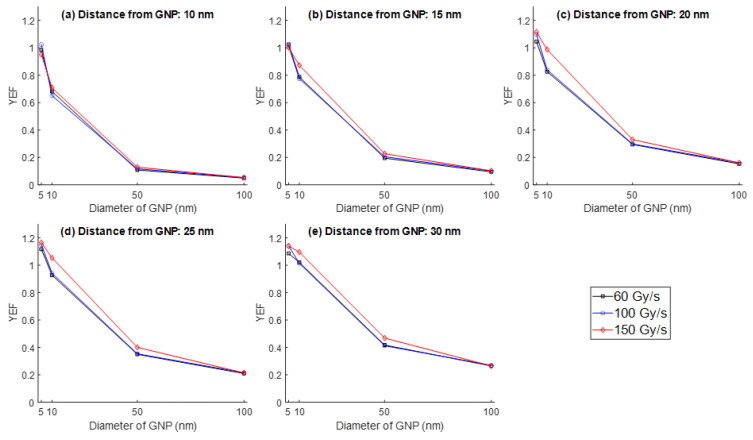
YEF vs. GNP size (5, 10, 50, and 100 nm) at distances of (**a**) 10 nm, (**b**) 15 nm, (**c**) 20 nm, (**d**) 25 nm, and (**e**) 30 nm from the surface of GNP at different UHDR (60, 100, and 150 Gy/s) for the electron energy of 100 keV.

The impact of GNP size on ROS generation was further analyzed across the four selected diameters (5, 10, 50, and 100 nm) at different radial distances (10–30 nm) from the nanoparticle surface. The YEF values for both 1 MeV and 100 keV electrons were plotted in Figure 4 and Figure 5, respectively.

For both electron energies, a clear inverse relationship was observed between GNP size and YEF. Smaller nanoparticles consistently yielded higher ROS enhancement, particularly for 5 nm GNPs, which exhibited peak YEF values under the 1 MeV, 60 Gy/s condition. At all distances, the YEF declined significantly with increasing GNP diameter, especially between 10 nm and 50 nm. For example, at 30 nm from the GNP surface under 1 MeV irradiation with a dose rate equal to 60 Gy/s (Figure 4e), YEF dropped from approximately 1.25 for 5 nm GNPs to less than 0.4 for 100 nm GNPs.

This trend was also present in the 100 keV electron case (Figure 5), although YEF values were generally lower compared to the high-energy scenario. Nevertheless, the results confirm that GNP size is a critical determinant of ROS amplification during FLASH irradiation.

## 4. Discussion

### 4.1. Dose Rate Effects on ROS Amplification

The results reveal that the FLASH dose rate affects ROS generation, but only under specific conditions. For 1 MeV electrons, lower dose rates (60 Gy/s) appeared to promote greater ROS yields in proximity to small GNPs, aligning with previous experimental findings that suggest FLASH irradiation can modulate radiolytic chemistry [43]. It was previously reported that as the mean dose rate of UHDR increased, ROS (H_2_O_2_) production decreased, warranting further optimization of dose rate parameters for UHDR radiotherapy [44].

These observations are also consistent with Chappuis et al. [3], who described dose rate–dependent differences in water radiolysis using Geant4-DNA. The reduced ROS yield at higher dose rates may be attributed to the saturation of radiolytic pathways or suppression of radical diffusion under rapid energy deposition conditions.

In contrast, low-energy electrons (100 keV) exhibited minimal sensitivity to UHDR conditions. In consideration of the fact that ROS can be induced through secondary electron interactions, this could be due to the shorter track lengths and reduced penetration depth of low-energy electrons, which limit their capacity to generate sufficient secondary electrons or promote extended ROS diffusion.

### 4.2. Role of GNP Size and Self-Absorption

The observed inverse relationship between GNP size and YEF can be attributed to several physical and chemical mechanisms. First, smaller GNPs allow a greater fraction of secondary electrons to escape their surfaces and interact with the surrounding medium. Conversely, larger GNPs exhibit self-absorption, whereby low-energy secondary electrons are reabsorbed or scattered internally, reducing the effective dose enhancement near the GNP surface [45,46,47,48].

Moreover, electron shadowing effects in larger GNPs have been reported to contribute to reduced radiolytic yields [49]. While the publications above recorded a reduction in ROS with increasing GNP size, another study found that ROS production increased with GNP size [50]. This discrepancy and the findings in our study highlight the importance of nanoparticle size in optimizing radiosensitization, especially under UHDR delivery, by balancing dose enhancement with energy escape efficiency.

In addition to size-dependent effects, it is important to note that aggregation of GNPs is frequently observed in biological environments due to factors such as protein corona formation, ionic interactions, or endocytosis. Aggregation can significantly alter the effective surface area exposed to radiation and modify the local electron transport conditions. Previous studies have shown that aggregated or clustered nanoparticles may reduce ROS yield compared to isolated particles, as closely packed GNPs can enhance self-absorption of low-energy electrons and limit the escape of secondary electrons into the surrounding medium [45,46,47,48]. Although our current simulation models a single, isolated GNP to provide a clean and quantifiable baseline for radiosensitization, future studies could incorporate multi-particle or clustered configurations [46] to better mimic physiological conditions and assess their impact on FLASH-RT outcomes.

### 4.3. Implications for FLASH Nanoparticle-Enhanced Radiotherapy

This study offers valuable computational insights into the combination of GNP-enhanced radiosensitization with UHDR electron therapy, a relatively unexplored domain in radiation oncology. The enhanced ROS generation observed under low UHDR and small GNP sizes suggests that careful selection of nanoparticle parameters and beam settings may maximize therapeutic benefits while maintaining the normal tissue-sparing effects of FLASH-RT.

However, it is important to note that this work focuses on single-pulse simulations and evaluates ROS yields near GNP (10–30 nm distance from the surface of GNP) at a fixed chemical time point (10 ns). Future studies should consider multiple pulse effects, pulse duration, biological endpoints (e.g., DNA damage), and experimental validation to build a more comprehensive model for clinical translation.

## 5. Conclusions

This study employed Geant4-DNA MC simulations to evaluate the impact of GNPs on ROS generation under UHDR electron-beam irradiation, mimicking FLASH-RT conditions. By comparing GNPs of various sizes (5–100 nm) with equivalent WNPs, the YEF was quantified as a measure of radiosensitization.

Results showed that small GNPs (5–10 nm) significantly enhanced ROS production under 1 MeV irradiation, particularly at lower dose rates (60 Gy/s), with YEF values reaching up to 1.25. In contrast, larger GNPs demonstrated limited enhancement regardless of dose rates, likely due to self-absorption effects. For 100 keV electrons, the dose rate had minimal influence on YEF.

These findings suggest that GNP size and beam energy play crucial roles in optimizing chemical enhancement during FLASH-RT. Although the MC simulation approximates complicated biological systems, limited to single-pulse irradiation and idealized conditions, this study offers computational insight into nanoparticle-mediated radiosensitization at UHDR.

This study has some limitations. The simulations assumed isolated GNPs, while aggregation is common in biological environments and may affect ROS yield. Only single-pulse irradiation was modeled, and biological endpoints such as DNA damage were not directly evaluated. These aspects will be addressed in future studies to enhance clinical relevance and experimental validation.

## Figures and Tables

**Table 1:** UHDR electron-beam parameters for Geant4-DNA Monte Carlo simulation.

Mode	Dose Per Pulse (Gy)	Dose Rate (Gy/s)
UHDR	0.6	60
UHDR	1	100
UHDR	1.5	150

## Data Availability

No new data were created.

## Abbreviations

The following abbreviations are used in this manuscript:

Abbreviation	Full Term
MC	Monte Carlo
Geant4-DNA	GEometry ANd Tracking version 4—DNA extension
UHDR	Ultrahigh Dose Rate
FLASH-RT	FLASH Radiotherapy
GNP	Gold Nanoparticle
WNP	Water Nanoparticle
ROS	Reactive Oxygen Species
YEF	Yield Enhancement Factor
DNA	Deoxyribonucleic Acid
DSB	Double-Strand Break
ISO	International Organization for Standardization
OH•	Hydroxyl Radical
H_2_O_2_	Hydrogen Peroxide
H•	Hydrogen Radical
H_3_O^+^	Hydronium Ion
OH^−^	Hydroxide Ion
MeV	Mega Electron Volt
keV	Kilo Electron Volt
CERN	European Organization for Nuclear Research

## References

[1] Vozenin M.C., Bourhis J., Durante M. (2022). Towards clinical translation of FLASH radiotherapy. Nat. Rev. Clin. Oncol..

[2] Favaudon V., Labarbe R., Limoli C.L. (2022). Model studies of the role of oxygen in the FLASH effect. Med. Phys..

[3] Chappuis F., Tran H.N., Jorge P.G., Zein S.A., Kyriakou I., Emfietzoglou D., Bailat C., Bochud F., Incerti S., Desorgher L. (2024). Investigating ultra-high dose rate water radiolysis using the Geant4-DNA toolkit and a Geant4 model of the Oriatron eRT6 electron linac. Sci. Rep..

[4] Chow C.L., Ruda H.E. (2023). Flash Radiotherapy: Innovative Cancer Treatment. Encyclopedia.

[5] Abolfath R., Grosshans D., Mohan R. (2020). Oxygen depletion in FLASH ultra-high-dose-rate radiotherapy: A molecular dynamics simulation. Med. Phys..

[6] Cao X., Zhang R., Esipova T.V., Allu S.R., Ashraf R., Rahman M., Gunn J.R., Bruza P., Gladstone D.J., Williams B.B. (2021). Quantification of oxygen depletion during FLASH irradiation in vitro and in vivo. Int. J. Radiat. Oncol. Biol. Phys..

[7] Dewey D.L., Boag J.W. (1959). Modification of the oxygen Effect When bacteria are Given Large pulses of radiation. Nature.

[8] Epp E.R., Weiss H., Djordjevic B., Santomasso A. (1972). The radiosensitivity of cultured mammalian cells exposed to single high intensity pulses of electrons in various concentrations of oxygen. Radiat. Res..

[9] Rosini G., Ciarrocchi E., D’Orsi B. (2025). Mechanisms of the FLASH effect: Current insights and advances. Front. Cell Dev. Biol..

[10] Zhou G. (2020). Mechanisms underlying FLASH radiotherapy, a novel way to enlarge the differential responses to ionizing radiation between normal and tumour tissues. Radiat. Med. Prot..

[11] Favaudon V., Caplier L., Monceau V., Pouzoulet F., Sayarath M., Fouillade C., Poupon M.-F., Brito I., Hupé P., Bourhis J. (2014). Ultrahigh dose-rate FLASH irradiation increases the differential response between normal and tumor tissue in mice. Sci. Transl. Med..

[12] Harrington K.J. (2019). Ultrahigh dose-rate RT: Next steps for FLASH-RT. Clin. Cancer Res..

[13] Loo B.W., Schuler E., Lartey F.M., Rafat M., King G.J., Trovati S., Koong A.C., Maxim P.G. (2017). (P003) Delivery of ultra-rapid flash radiation therapy and demonstration of normal tissue sparing after abdominal irradiation of mice. Int. J. Radiat. Oncol..

[14] Vozenin M.-C., De Fornel P., Petersson K., Favaudon V., Jaccard M., Germond J.-F., Petit B., Burki M., Ferrand G., Patin D. (2019). The advantage of Flash RT confirmed in mini-pig and cat-cancer patients. Clin. Cancer Res..

[15] Mascia A.E., Daugherty E.C., Zhang Y., Lee E., Xiao Z., Sertorio M., Woo J., Backus L.R., McDonald J.M., McCann C. (2023). Proton FLASH Radiotherapy for the Treatment of Symptomatic Bone Metastases The FAST-01 Nonrandomized Trial. JAMA Oncol..

[16] (2015). Nanotechnologies—Vocabulary—Part 1: Core Terms.

[17] Nguyen N.H.A., Falagan-Lotsch P. (2023). Mechanistic Insights into the Biological Effects of Engineered Nanomaterials: A Focus on Gold Nanoparticles. Int. J. Mol. Sci..

[18] Chen Y., Yang J., Fu S., Wu J. (2020). Gold nanoparticles as radiosensitizers in cancer radiotherapy. Int. J. Nanomed..

[19] Bardane A., Maalej N., Chakir E.M., Ibrahmi E.M.A. (2024). Gold nanoparticle effect on dose and DNA damage enhancement in the vicinity of gold nanoparticles. Nucl. Anal..

[20] Leenhouts H.P., Chadwick K.H. (1978). The crucial role of DNA double-strand breaks in cellular radiobiological effects. Advances in Radiation Biology.

[21] Misawa M., Takahashi J. (2011). Generation of reactive oxygen species induced by gold nanoparticles under X-ray and UV irradiations. Nanomedicine.

[22] Butterworth K.T., Coulter J.A., Jain S., McMahon S.J., Schettino G., Prise K.M., Currell F.J., Hirst D.G. (2010). Evaluation of cytotoxicity and radiation enhancement using 1.9 nm gold particles: Potential application for cancer therapy. Nanotechnology.

[23] Hainfeld J.F., Smilowitz H.M., O’Connor M.J., Dilmanian F.A., Slatkin D.N. (2013). Gold nanoparticle imaging and radiotherapy of brain tumors in mice. Nanomedicine.

[24] Hullo M., Grall R., Perrot Y., Mathé C., Ménard V., Yang X., Lacombe S., Porcel E., Villagrasa C., Chevillard S. (2021). Radiation enhancer effect of platinum nanoparticles in breast cancer cell lines: In vitro and in silico analyses. Int. J. Mol. Sci..

[25] Chow J.C. (2024). Biophysical insights into nanomaterial-induced DNA damage: Mechanisms, challenges, and future directions. AIMS Biophysics..

[26] Lo C.-Y., Tsai S.-W., Niu H., Chen F.-H., Hwang H.-C., Chao T.-C., Hsiao I.-T., Liaw J.-W. (2023). Gold-Nanoparticles-Enhanced Production of Reactive Oxygen Species in Cells at Spread-Out Bragg Peak under Proton Beam Radiation. ACS Omega.

[27] Chappuis F., Tran H.N., Zein S.A., Bailat C., Incerti S., Bochud F., Desorgher L. (2023). The general-purpose Geant4 Monte Carlo toolkit and its Geant4-DNA extension to investigate mechanisms underlying the FLASH effect in radiotherapy: Current status and challenges. Phys. Med..

[28] Sakata D., Kyriakou I., Okada S., Tran H.N., Lampe N., Guatelli S., Bordage M.-C., Ivanchenko V., Murakami K., Sasaki T. (2018). Geant4-DNA track-structure simulations for gold nanoparticles: The importance of electron discrete models in nanometer volumes. Med. Phys..

[29] Engels E., Bakr S., Bolst D., Sakata D., Li N., Lazarakis P., McMahon S.J., Ivanchenko V., Rosenfeld A.B., Incerti S. (2020). Advances in modelling gold nanoparticle radiosensitization using new Geant4-DNA physics models. Phys. Med. Biol..

[30] Lechtman E., Mashouf S., Chattopadhyay N., Keller B.M., Lai P., Cai Z., Reilly R.M., Pignol J.-P. (2013). A Monte Carlo-based model of gold nanoparticle radiosensitization accounting for increased radiobiological effectiveness. Phys. Med. Biol..

[31] Lechtman E., Chattopadhyay N., Cai Z., Mashouf S., Reilly R., Pignol J.P. (2011). Implications on clinical scenario of gold nanoparticle radiosensitization in regards to photon energy, nanoparticle size, concentration and location. Phys. Med. Biol..

[32] Tsai M.-Y., Tian Z., Qin N., Yan C., Lai Y., Hung S.-H., Chi Y., Jia X. (2020). A new open-source GPU-based microscopic Monte Carlo simulation tool for the calculations of DNA damages caused by ionizing radiation-Part I: Core algorithm and validation. Med. Phys..

[33] Chow J.C.L., Leung M.K.K., Fahey S., Chithrani D.B., Jaffray D.A. (2012). Monte Carlo simulation on low-energy electrons from gold nanoparticle in radiotherapy. J. Phys. Conf. Ser..

[34] Lin Y., McMahon S.J., Scarpelli M., Paganetti H., Schuemann J. (2014). Comparing gold nano-particle enhanced radiotherapy with protons, megavoltage photons and kilovoltage photons: A Monte Carlo simulation. Phys. Med. Biol..

[35] Tran H.N., Karamitros M., Ivanchenko V.N., Guatelli S., McKinnon S., Murakami K., Sasaki T., Okada S., Bordage M.C., Francis Z. (2016). Geant4 Monte Carlo simulation of absorbed dose and radiolysis yields enhancement from a gold nanoparticle under MeV proton irradiation. Nucl. Inst. Meth. B..

[36] Peukert D., Kempson I., Douglass M., Bezak E. (2020). Gold nanoparticle enhanced proton therapy: A Monte Carlo simulation of the effects of proton energy, nanoparticle size, coating material, and coating thickness on dose and radiolysis yield. Med. Phys..

[37] Villagrasa C., Francis Z., Incerti S. (2011). Physical models implemented in the GEANT4-DNA extension of the GEANT-4 toolkit for calculating initial radiation damage at the molecular level. Radiat. Prot. Dosim..

[38] Incerti S., Suerfu B., Xu J., Ivantchenko V., Mantero A., Brown J.M.C., Bernal M.A., Francis Z., Karamitros M., Tran H.N. (2016). Simulation of Auger electron emission from nanometer-size gold targets using the Geant4 Monte Carlo simulation toolkit. Nucl. Instrum. Methods Phys. Res. Sect. B.

[39] Tran H.N., Archer J., Baldacchino G., Brown J.M.C., Chappuis F., Cirrone G.A.P., Desorgher L., Dominguez N., Fattori S., Guatelli S. (2024). Review of chemical models and applications inGeant4-DNA: Report from the ESA BioRad III Project. Med. Phys..

[40] Jorge P.G., Grilj V., Bourhis J., Vozenin M.-C., Germond J.-F., Bochud F., Bailat C., Moeckli R. (2022). Technical note: Validation of an ultrahigh dose rate pulsed electron beam monitoring system using a current transformer for FLASH preclinical studies. Med. Phys..

[41] Jaccard M., Durán M.T., Petersson K., Germond J.-F., Liger P., Vozenin M.-C., Bourhis J., Bochud F., Bailat C. (2018). High dose-perpulse electron beam dosimetry: Commissioning of the Oriatron eRT6 prototype linear accelerator for preclinical use. Med. Phys..

[42] Zhao X., Liu R., Zhao T., Reynoso F.J. (2021). Quantification of gold nanoparticle photon radiosensitization from direct and indirect effects using a complete human genome single cell model based on Geant4. Med. Phys..

[43] Thomas W., Sunnerberg J., Reed M., Gladstone D.J., Zhang R., Harms J., Swartz H.M., Pogue B.W. (2023). Proton and electron UHDR isodose irradiations produce differences in reactive oxygen species yields. Int. J. Radiat. Ocol. Biol. Phys..

[44] Sunnerberg J.P., Zhang R., Gladstone D.J., Swartz H.M., Gui J., Pogue B.W. (2023). Mean dose rate in ultra-high dose rate electron irradiation is a significant predictor for O_2_ consumption and H_2_O_2_ yield. Phys. Med. Biol..

[45] Poignant F., Charfi H., Chan C.-H., Dumont E., Loffreda D., Testa É., Gervais B., Beuve M. (2020). Monte Carlo simulation of free radical production under keV photon irradiation of gold nanoparticle aqueous solution. Part. I: Global primary chemical boost. Radiat. Phys. Chem..

[46] Santiago C.A., Chow J.C.L. (2023). Variations in Gold Nanoparticle Size on DNA Damage: A Monte Carlo Study Based on a Multiple-Particle Model Using Electron Beams. Appl. Sci..

[47] Chow J.C.L., Santiago C.A. (2023). DNA Damage of Iron-Gold Nanoparticle Heterojunction Irradiated by kV Photon Beams: A Monte Carlo Study. Appl. Sci..

[48] Jabeen M., Chow J.C.L. (2021). Gold Nanoparticle DNA Damage by Photon Beam in a Magnetic Field: A Monte Carlo Study. Nanomaterials.

[49] Peukert D., Kempson I., Douglass M., Bezak E. (2019). Gold Nanoparticle Enhanced Proton Therapy: Monte Carlo Modeling of Reactive Species’ Distributions Around a Gold Nanoparticle and the Effects of Nanoparticle Proximity and Clustering. Int. J. Mol. Sci..

[50] Antunes J., Rabus H., Mendes F., Paulo A., Sampaio J.M. (2025). Chemical mechanism in gold nanoparticles radiosensitization: A Monte Carlo simulation study. Radiat. Phys. Chem..

